# Modulation of amygdala and hippocampus during anxiety by heart and middle frontal gyrus

**DOI:** 10.1093/cvr/cvaf007

**Published:** 2025-01-21

**Authors:** Gert Pfurtscheller, Beate Rassler, Alberto Porta, Gerhard Schwarz, Maciej Kaminski, Klaus Pfurtscheller, Wolfgang Klimesch

**Affiliations:** Institute of Neural Engineering, Graz University of Technology, Graz, Austria; Carl-Ludwig-Institute of Physiology, University of Leipzig, Leipzig, Germany; Department of Biomedical Sciences for Health, University of Milan, Milan, Italy; Department of Cardiothoracic, Vascular Anesthesia and Intensive Care, IRCCS Policlinico San Donato, Milan, Italy; Department of Anaesthesiology and Intensive Care Medicine, Medical University of Graz, Graz, Austria; Faculty of Physics, University of Warsaw, Warsaw, Poland; Paediatric Intensive Care and Burn Unit, University Childrens Hospital and Medical University of Graz, Graz, Austria; Centre of Cognitive Neuroscience, University of Salzburg, Salzburg, Austria

## 1. Introduction

The cardiac RR interval (RRI) rhythm consists of various frequency components. While oscillations around 0.1 Hz are relatively well studied, primarily due to their vascular and central origin, higher frequency cardiac oscillations are less explored but warrant attention due to their potential coupling with neural rhythms.^1^ In the context of brain–body interactions, two distinct brain states can be identified: a resting/relaxed state and an anxious/panicked state.^2^

## 2. Anxiety and emotional breathing oscillations at ∼0.32 Hz

**Figure 1:**
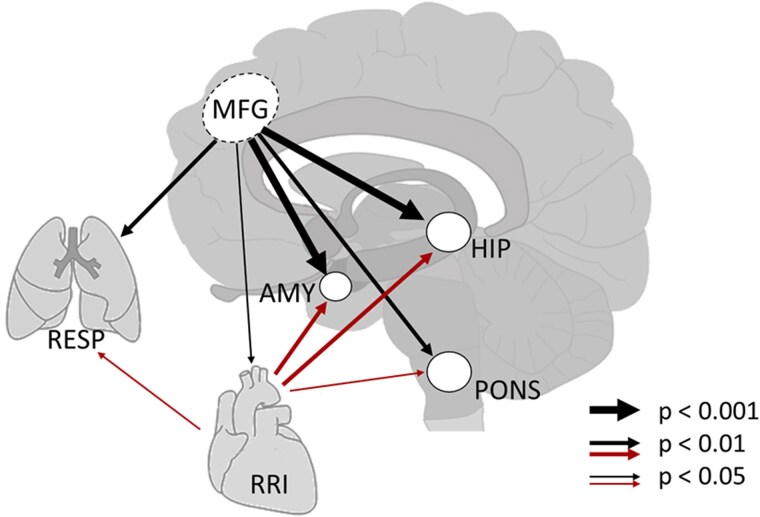
The image provides a visual summary of our key research findings. It highlights the specific brain regions studied, including cortical (MFG), subcortical (AMY, and HIP), and brainstem (pons caudal) areas, as well as the relevant body systems (lung and heart). The figure also depicts the strength and direction of coupling in the 0.2–0.4 Hz frequency band. Arrows indicate the significance levels of directed coupling: highly significant (*P* < 0.001, *P* < 0.01) and significant (*P* < 0.05). Notably, the image emphasizes two primary findings: downward-directed activations from MFG to AMY, HIP, respiration and cardiac system, and upward-directed activation originating from the cardiac system.

Anxiety with a breathing rate (BR) between 0.24 and 0.37 Hz is prevalent in patients with medically intractable epilepsy during intracranial EEG (iEEG) recordings,^3,4^ as well as in patients undergoing MRI scans for serious conditions, such as cancer. Remarkably, a similar breathing pattern (∼0.32 Hz or ∼19 bpm) is frequently observed in healthy volunteers experiencing MRI-related anxiety.^5^ This observation is intriguing because 0.32 Hz corresponds to one of the three preferred breathing frequencies in Klimesch’s binary hierarchy brain–body model.^6^ Although our healthy MRI participants were not given specific instructions regarding their breathing pattern (i.e. nasal or oral), it can be assumed that nasal breathing predominated. This assumption is supported by the highly significant coupling between the BOLD signal from the middle frontal gyrus (MFG) and respiration (see *Figure 1*).^7,8^ An interesting aspect is also that nasal breathing is essential for neonates’ survival.

## 3. Information flow during brain–body interaction?

The two main brain structures of the limbic system related to anxiety processing are the hippocampus (HIP) and the multinuclear amygdala (AMY) that can enter into interactions with important body signals, such as RRI and respiration. We therefore asked, whether the activation of limbic structures can be detected during spontaneous breathing at ∼0.32 Hz in healthy people with MRI-related anxiety.

ECG and respiration were recorded from 23 healthy participants along with BOLD signals using a 3T scanner at a sampling rate of 1/871 ms (1.15 Hz). All participants gave informed written consent to the protocol of the study, which was approved by the local Ethics Committee at the University of Graz (number GZ.39/75/63ex2013/14) and was therefore performed in accordance with the ethical standards laid down in the 1964 Declaration of Helsinki. From the ECG (thoracic lead), the RRI signal was extracted. Furthermore, we recorded respiration (RESP) via a chest belt, three BOLD signals from the left cortex (MFG, HIP, and AMY) and one BOLD signal from the brainstem [left caudal pons (pons)]. Notably, no standard pre-processing of the BOLD signals was performed. The averages for RRI, BR, age, and anxiety scores (AS) are: frequency of RRI = 0.10 ± 0.01 Hz, BR = 0.32 ± 0.03 Hz, age = 24.3 ± 2.3 years, and AS = 24.6 ± 2.5. Using multivariate autoregressive modelling of the RRI, breathing, and four BOLD signals, the Directed Transfer Function was computed, allowing for the assessment of directed coupling within the 0.2–0.4 Hz frequency range.^9^ Surrogate data and bootstrap approach were used for statistical analysis. For further details, see Pfurtscheller *et al*.^7^*Figure 1* displays the summarized results of such a study on healthy participants with MRI-related anxiety focused on the frequency band 0.2−0.4 Hz. Besides the predominant downward-directed activations from the MFG towards limbic structures, RRI and breathing signals are also modulated. This suggests a significant role of the MFG in controlling cognitive and emotional processes. It is important to note that our data do not represent a conventional resting or relaxed state, as achieving a brain state completely free of anxiety during MRI studies is hardly possible.^7^

## 4. Information flow from cortex to AMY and HIP

The most impressive finding of our study is the highly significant (*P* < 0.001) information flow from the MFG to the HIP and AMY in the 0.2–0.4 Hz band during MRI-related anxiety. As illustrated in the two black arrows in *Figure 1*, the major flow appears to be unidirectional from the MFG to the limbic structures, with feedback being negligible. The significant projection from the MFG to RESP also indicates a strong downward-directed activation that overrides the intrinsic drive of the breathing signal.

The emotional BR of healthy MRI participants experiencing scanner-related anxiety ranged from 0.28 to 0.36 Hz, which corresponds to the BR of 0.24–0.37 Hz observed in patients with epilepsy during intracranial EEG recordings and nasal breathing.^3,4^ In the former case, the BOLD oscillations in the MFG project onto the HIP and AMY and activate them. In the latter (iEEG), breathing-entrained local field potential oscillations drive theta oscillations in the HIP, AMY, and piriform cortex.

## 5. Information flow from cardiac RRI oscillations to AMY and HIP

Another novel finding is the significant (*P* < 0.01) projection of RRI oscillations to BOLD oscillations originating in the HIP and AMY (*Figure 1*, red arrows). *Figure 1* also shows that respiration is strongly involved in these flow interactions, with a dominant flow from RRI to respiration known as negative RSA.^8^ A recent study on the coupling of rhythms in EEG and ECG reported a significant dominance of heart-to-brain effects over brain-to-heart effects, particularly in the theta band during breathing with BR = 0.27 ± 0.05 Hz.^1^ This finding is confirmed by the higher proportion of afferent fibres of the vagus nerve (∼80%) compared with efferent fibres (∼20%) between the heart and the brain. This agrees with our results, as the spectral breathing peak of the cardiac signal in our data was between 0.28 and 0.36 Hz, which is also supported by another study.^10^ The projection of RRI to BOLD oscillations in the HIP and AMY suggests that the cardiac system is linked to various limbic structures capable of modulating their activity within the 0.2–0.4 Hz band. Notably, this connection between the cardiac system (RRI) and the HIP and AMY is bidirectional and is characterized by strong feedback. This reinforces the assumption that cardiac oscillations significantly contribute to BOLD signal fluctuations across different frequency ranges.

## Data Availability

Data available on request. We like to thank David Fink and Andreas Schwerdtfeger, University Graz, for data acquisition.
